# Comparison of zinc bioavailability in zinc-glycine and zinc-methionine chelates for broilers fed with a corn-soybean meal diet

**DOI:** 10.3389/fphys.2022.983954

**Published:** 2022-11-17

**Authors:** Xiaohui Chen, Chao He, Keying Zhang, Jianping Wang, Xuemei Ding, Qiufeng Zeng, Huanwei Peng, Jie Bai, Li Lv, Yue Xuan, Shiping Bai

**Affiliations:** Animal Nutrition Institute, Feed Engineering Research Centre of Sichuan Province, Sichuan Agricultural University, Chengdu, China

**Keywords:** organic zinc, bioavailability, pancreas metallothionein, zinc accumulation, chicken

## Abstract

The objective of this study was to compare the bioavailability of zinc (Zn) from zinc-glycine (Zn-Gly) and zinc-methionine (Zn-Met) as compared with zinc sulfate (ZnSO_4_) used as a standard in broilers. A total of 1,200 one-day-old male broilers (Cobb 500) were randomly allotted to one of 10 treatments with eight replicate cages of 15 birds each. The broilers were fed a corn-soybean meal basal diet (containing 26.46 mg Zn/kg; control) or the basal diet added with 40, 80, and 120 mg Zn/kg as Zn-Gly, Zn-Met, or ZnSO_4_ for 14 days. The relative bioavailability value (RBV) was calculated based on multiple linear regression slope ratios of Zn concentrations in tibia and pancreas, pancreas metallothionein (MT) concentration, and pancreas *MT* mRNA abundance on added Zn intake. When comparing the control with all Zn-supplemented treatments, Zn addition did not significantly affect average feed intake and bodyweight gain during days 1–14 (*p* > 0.10). However, Zn concentrations in the tibia, pancreas, and liver and pancreas MT concentration and *MT* mRNA abundance increased in all Zn-supplemented treatments compared with the control (*p* < 0.05), and these indices increased linearly (*p* < 0.001) with increasing added Zn levels on days 7 and 14. The RBV of Zn as Zn-Met was similar to that as Zn-Gly or ZnSO_4_ (*p* > 0.40) on days 7 and 14, based on tibia and pancreas Zn. In contrast, on days 7 and 14, the RBVs of Zn were in the following order: Zn-Met > Zn-Gly > ZnSO_4_ (*p* < 0.05), based on pancreas MT concentration. The bioavailable Zn from Zn-Met was 1.20 or 1.25 times that from Zn-Gly on day 7 or 14, respectively, evaluated by pancreas MT content. The RBV of Zn as Zn-Met was similar to that as Zn-Gly or ZnSO_4_ on day 7, whereas it was higher than that as Zn-Gly or ZnSO_4_ on day 14, based on pancreas *MT* mRNA abundance. In conclusion, Zn-Met had higher bioavailable Zn than Zn-Gly for the starter broilers fed with the corn-soybean meal diet, using pancreas MT concentration as the response criterion.

## Introduction

Zinc (Zn) is one of the essential trace minerals for the growth and development of broilers (Baltaci et al., 2018). It plays an important role in protein and DNA synthesis and the metabolism of lipids and carbohydrates because Zn ion is an essential cofactor of some metalloenzymes involved in these biological processes (McCall et al., 2000). The National Research Council (1994) reported the zn requirement of the starter broiler was 40 mg/kg in the purified or semipurified diet based on the growth performance of broilers. However, when the corn-soybean meal is used, the Zn requirement is 60 mg of Zn/kg of diet for the starter broiler assessed by the tibia Zn content (Wedekind et al., 1992). Huang et al. (2007) also found that the optimal Zn requirement of chicks from hatch to 21 days of age was 59.15 mg/kg for pancreas Zn and 61.70 mg/kg for bone Zn in a corn-soybean meal diet; however, the optimal dietary Zn level of the starter broilers was 84 mg/kg assessed by the serum 5′-nucleotidase activity and pancreas Zn transporter 2 mRNA level. Hence, the differences in the Zn requirement for the starter broiler partly lie in the differences in the basal diet and the choice of responsive parameters.

Recently, considerable attention has been focused on the bioavailability of Zn from different supplemental Zn sources for broiler chickens. Supplemental Zn must be used in broiler diets to optimize the biological responses of Zn because the Zn concentration in the natural feedstuff ingredients does not meet the requirement of broiler chickens (Shao et al., 2019). The growth performance and Zn content in different tissues are the biomarkers to assess the Zn bioavailability from different Zn sources when the dietary Zn level is below the Zn requirement of chickens (Wedekind and Baker, 1990; Batal et al., 2001). Bioavailability of Zn from Zn-methionine chelate compared with Zn sulfate (ZnSO_4_; set as 100%) are 124% and 177%, respectively, assessed by weight gain and tibia Zn of broilers (Wedekind et al., 1992). A disadvantage of growth rate assays lies in the fact that the method requires the use of semipurified diets, which may yield results not entirely applicable when practical diets containing natural ingredients are fed. Star et al. (2012) reported that the relative bioavailability of one Zn-proteinate complex was 1.64 compared with zinc sulfate as a reference zinc source (1.00) using tibia Zn content as a response parameter. However, the growth performance is not sensitive to evaluate the Zn bioavailability since it can be affected by many nutrient factors in practical diets (Wedekind et al., 1992; Sandoval et al., 1997). Recent studies have demonstrated that the activities and transcriptional expression of Zn regulatory enzymes, such as metallothionein (MT), are sensitive responsive indicators to evaluate the available Zn from different Zn sources in animals (Cousins et al., 2003; Huang et al., 2009; Suo et al., 2015).

The chemical form of supplemental Zn source affects the Zn bioavailability. Zn is added to broiler diets usually as inorganic feed-grade ZnSO_4_, Zn oxide, Zn-amino acid chelate, or Zn-proteinate complex. Organic Zn sources have been used increasingly due to their potentially high Zn bioavailability, although there are some conflicting findings (Kidd et al., 1996; Huang et al., 2009; Salim et al., 2010; Star et al., 2012). It is well established that an inorganic metal salt has a different absorption level than a metal-amino acid chelate (Ashmead, 2012). Even among metal-amino acid chelates, absorption rates vary according to the ligand, stability constants, molecular weights, *etc.* (Ashmead, 2012). Huang et al. (2009) found that the Zn bioavailability from Zn-amino acid or Zn-proteinate complex was closely related to their chelation strength, the value representing stability constant, in starter broilers. However, little research has been carried out to compare the Zn bioavailability from different Zn-amino acid chelates in broilers under the same conditions. The objective of this study was to compare the Zn bioavailability from Zn-glycine (Zn-Gly) and Zn-methionine chelate (Zn-Met) as compared with ZnSO_4_ used as a standard in the corn-soybean meal basal diet for the starter broiler chickens.

## Materials and methods

### Animals and diets

A total of 1,200 one-day-old male broilers (Cobb 500) were randomly allotted to one of 10 treatments with eight replicate cages of 15 chicks each. Dietary treatments included the corn-soybean meal basal diet (control) and the basal diet supplemented with 40, 80, or 120 mg Zn/kg from ZnSO_4_, Zn-Gly, or Zn-Met. The basal diet (Table 1) was formulated to meet or exceed the requirements of starter broilers (day 1–14) as recommended by the Feeding Standard of Chicken (Ministry of Agriculture of the People’s Republic of China, 2004), except for Zn. The basal diet contained 26.46 mg Zn/kg by analysis. Supplemental ZnSO_4_ was provided as ZnSO_4_•7H_2_O (reagent grade, purity 99.95%), purchased from Chendu Xilong Biochemical Company (Chengdu, China). The Zn-Gly and Zn-Met chelates contained 19.00% and 23.75% Zn, respectively, purchased from Sichuan Chelota Group (Mianyang, China). In these chelates, the glycine or methionine molecule is bound with Zn^2+^ by oxygen and nitrogen atoms (Chang et al., 2022; Wang et al., 2022). In the basal diet, calcium carbonate and dicalcium phosphate (Chendu Xilong Biochemical Company, Chengdu, China) were reagent-grade, and the purity of these compounds was more than 99.00%. Each Zn source was premixed with maize starch to the same weight and then was added to the respective diet based on its analyzed Zn concentration. Variable amounts of glycine and methionine were added to the respective experimental diets according to the amounts of glycine and methionine from supplemental Zn-glycine and Zn-methionine chelates so as to balance the glycine and methionine in each experimental diet. Dietary Zn concentrations are presented in Table 2. Drinking water contained 30.2 μg Zn/L.

**TABLE 1:** Ingredient and nutrient composition of the basal diet (as-fed basis).

Ingredient	Value (%)	Calculated nutrient level	Value (%)
Corn	54.76	Metabolizable energy (MJ/kg)	12.44
Soybean meal	35.40	Crude protein	21.28
Pea gluten meal	2.00	Lysine	1.12
Soybean oil	4.00	Methionine	0.56
CaCO_3_ ^a^	1.15	Methionine + Cystine	0.91
CaHPO_4_•2H_2_O^a^	1.80	Calcium^b^	1.00 (1.00)
DL-methionine	0.24	Nonphytate phosphosrus^b^	0.45 (0.46)
NaCl	0.03	Zinc^b^ (mg/kg)	24.12 (26.46)
Choline chloride	0.12		
Mineral mix^c^	0.20		
Vitamin mix^d^	0.03		

^a^ Reagent grade.

^b^ Calculated values with analyzed values in parentheses.

^c^ Provided per kg of complete diet: 80 mg of Fe (FeSO_4_·H_2_O), 60 mg of Mn (MnSO_4_·H_2_O), 8 mg of Cu (CuSO_4_·5H_2_O), 0.35 mg of I (KI), and 0.30 mg of Se (Na_2_SeO_3_).

^d^ Provided per kg of complete diet: 12,000 IU, vitamin A (retinyl palmitate); 3,000 IU, cholecalciferol; 7.5 IU, vitamin E (DL-tocopherol acetate); 0.6 mg of thiamine; 4.8 mg of riboflavin; 1.8 mg of pyridoxine; 7.5 mg of pantothenic acid; 0.15 mg of folic acid; 10.5 mg of nicotinic acid; and 0.18 mg of biotin.

**TABLE 2:** Analyzed Zn concentrations in different diets for starter broilers (as-fed basis).

Zn source	Added Zn (mg/kg)	Analyzed Zn^a^ (mg/kg)
Control	0	26.46
ZnSO_4_	40	71.57
	80	120.2
	120	150.0
Zn-methionate	40	66.14
	80	117.4
	120	155.2
Zn-glycinate	40	68.36
	80	117.3
	120	155.1

^a^ Values based on triplicate determinations of diet samples.

The broiler chickens were fed different treatment diets for 14 days. All diets were offered in a mash form. All birds were housed in electrically heated, thermostatically controlled cages (100 × 100 × 50 cm) with a 16-h light/8-h dark photocycle. The chicks were allowed *ad libitum* access to the experimental diets and water. The ambient temperature was gradually decreased by 0.5°C per day from 32°C on day 1 to 25°C on day 14, and the relative humidity of the house was kept at 60%.

### Growth performance

On day 1, the body weight (BW) of birds was measured, and on days 7 and 14, the BW and feed consumption were measured for each replicate cage after 8-h fasting. Mortality was recorded during the experimental period. The body weight gain (BWG), average feed intake (AFI), and the ratio of feed consumption and BWG (FCR) were calculated during days 1–7, 8–14, and 1–14.

### Sample collection

On days 7 and 14, two chicks were selected from each replicate cage according to average body weight and were sacrificed by cervical dislocation after 12-h fasting. The pancreas, left lobe of the liver, and the left tibia were excised and frozen at −20°C for Zn or MT protein analysis. Another subsample of the pancreas was frozen in liquid nitrogen for MT mRNA analysis.

### Zn concentrations in different tissues

The concentrations of Zn in the diets and tissues were determined by flame atomic absorption spectrometry (ContrAA700, Jena Company, Germany) as described by Zhao et al. (2019). Approximately 1.0 g of the pancreas or liver or 0.5 g of diet samples was digested with 10 ml of the mixed acid of HNO_3_ and HF (4:1, v:v) using a microwave digestion system (MARS6, CEM Corporation, Matthews, NC, United States). The digestion procedure was as follows: the temperature was increased to 120°C within 5 min and maintained for 10 min and then increased up to 180°C within the next 5 min and maintained for 90 min. After digestion, the digestion tubes were taken out, and the mixture acid was evaporated to 0.5 ml at 160°C using a heater (Model BHW-09Y, Botonyc, Beijing, China). After cooling, the digested solution was diluted to 50 ml volume with 0.5% nitric acid for Zn analysis. The frozen tibia samples were boiled for 10 min in deionized water, and all soft tissues were removed. The tibia samples were then dried at 105°C and ashed at 550°C for 8 h in a muffle furnace. The tibia ash was dissolved in 0.5% nitric acid for Zn analysis. For quality control, a standard reference of wheat powder purchased from National Institute of Standards and Technology (Beijing, China) was included in each batch of analysis to verify the Zn analysis validation.

### Pancreas MT concentration

The concentration of MT in pancreas was determined using a chicken MT ELISA kit (Nanjing Jiancheng Bioengineering Institute, Nanjing, China) following the manufacturer’s instructions. Briefly, approximately 0.2 g of the pancreas was transferred to a 5-ml tube containing 2 ml of lysis buffer (50 mM Tris-Cl, 1 mM EDTA, and 1 mM β-mercaptoethanol, pH 7.5). The pancreas samples were homogenized on ice with a homogenizer (Bio-Gen PRO200 Homogenizer, Pro Scientific Co., Oxford, CT, United States) for 30 s at 300 × rpm. This was repeated in 30 s pulses alternating with 30 s on ice twice. The lysates were then centrifuged at 13,000 × g at 4°C for 30 min, and the supernatant was collected as sample solution for determining the concentrations of MT and total protein. For the MT analysis, 50 μl of the MT standard solution or sample solution was incubated for 2 h at room temperature in each well (containing the immobilized murine monoclonal antibody against chicken MT). A 50 μl volume of the antibody–enzyme (horseradish peroxidase) conjugate solution was then incubated in the wells at 37°C for 30 min. Between the aforementioned steps, the plate was emptied and washed three times with a washing buffer. After drying, 50 μl volume of substrate mixture tetramethyl benzidine was pipetted into each well. The enzymatic reaction took place for 10 min at 37°C and was then stopped by adding 50 μl of a 2 M H_2_SO_4_ solution. Using a blank well as a reference (zero optical density), the optical density of each well under 450 nm wavelength was measured. Protein concentration was quantified with Bradford reagent (Sigma-Aldrich, United States) using an Infinite M200Pro multimode plate reader and Magellan software (Tecan, United States).

### Pancreas *MT* mRNA abundance

Total RNA was extracted from frozen pancreas samples using RNAiso plus reagent (Takara, Dalian, China). The quality of extracted RNA was detected by 1% agarose gel electrophoresis. The purity and concentration of RNA were determined using a nucleic acid protein analyzer (NanoDrop 2000, Thermo Scientific, United States). First-strand cDNA was synthesized from 200 ng of total RNA with a PrimeScript TM RT reagent kit (Takara, Dalian, China). The transcription procedure was as follows: 37°C for 120 min and 85°C for 5 min. Real-time PCR was performed in duplicate in 20 μl system that contained 10 μl SYBR Green PCR mix (TaKaRa, China), 2 μl of diluted cDNA, 0.8 μl of 10 μM each primer, and 6.4 μl of PCR-grade water using a 7,900 Fluorescence quantitative PCR (Applied Biosystems, United States). The primer sequences were as follows: *MT* (forward) 5′-AAG​GGC​TGT​GTC​TGC​AAG​G-3′, (reverse) 5′-CTT​CAT​CGG​TAT​GGA​AGG​TAC-3’; and *β-actin* (forward) 5′- GAG​AAA​TTG​TGC​GTG​ACA​TCA -3′, (reverse) 5′-CCT​GAA​CCT​CTC​ATT​GCC​A-3’. Real-time PCR reaction condition was in the following: 95°C for 30 s and 40 cycles of 95°C for 5 s plus 60°C for 34 s. The dissolution procedure was as follows: 95°C for 15 s, 60°C for1 min, and 95°C for 15 s. The *β-actin* was selected as the reference gene (Bai et al., 2020), and the *MT* mRNA expression was calculated using the 2^−ΔΔCt^ method (Livak and Schmittgen, 2001).

### Statistical analysis

Data were statistically analyzed using the general linear model procedure using SAS 9.2 software (SAS Institute Inc., Cary, NC, United States). To compare all Zn-supplemented treatments with the control, the data were analyzed using the one-way single degree of freedom contrast method (Suo et al., 2015). The data of all treatments except for the control were analyzed by two-way ANOVA, and the statistical model included Zn source, added Zn level, and their interaction. Linear responses of Zn concentrations in the tibia, liver, and pancreas, pancreas MT content, and pancreas *MT* mRNA abundance to the graded supplemental Zn level were evaluated using the orthogonal polynomial coding in the CONTRAST statement. Relative bioavailability value (RBV) of Zn was determined using Zn sulfate as a standard by means of multiple linear regression and slope ratio methodology (Suo et al., 2015). The liver Zn concentration was not significantly correlated to supplemental Zn intake for broilers on days 7 and 14, and it was consequently not used to calculate RBV estimate. Standard errors were calculated for each regression coefficient as described by Littell et al. (1997). The replicate cage was served as the experimental unit. Significant differences among means were tested by the least square difference method. For all statistical analyses, significance was declared at *p* < 0.05, and tendency was declared at 0.05 < *p* < 0.10.

## Results

### Growth performance

The effects of supplemental Zn source and Zn level on the growth performance of broilers are shown in Table 3. Overall, Zn supplementation decreased (*p* < 0.05) the AFI and FCR but did not significantly affect the BWG of broilers during days 1–7 compared with the control (*p* > 0.1). In all Zn-added treatments, Zn-Met and Zn-Gly addition significantly increased (*p* < 0.05) the BWG compared with ZnSO_4_ during days 1–7, whereas no significant difference was observed between the Zn-Met and Zn-Gly treatments. Meanwhile, they also tended to increase the BW on day 7 compared with the ZnSO_4_ treatment (*p* = 0.05). Irrespective of added Zn source, addition of 80 mg Zn/kg tended to increase the AFI compared with 40 mg Zn/kg treatment (*p* = 0.05). For ZnSO_4_ treatments, addition of 80 or 120 mg Zn/kg increased FCR compared with 40 mg Zn/kg addition group (*p* < 0.04). The addition of 80 mg Zn/kg also increased FCR compared with the 40 mg Zn/kg addition group for the Zn-Met treatment (*p* = 0.04), while addition of 120 mg Zn/kg decreased it compared with the 80 mg Zn/kg addition group (*p* = 0.03). However, added Zn level did not significantly affect FCR for the Zn-Gly treatment during days 1–7 (*p* > 0.4), and these ratios were lower than that in 80 or 120 mg Zn/kg as the ZnSO_4_ addition group (*p* < 0.05).

**TABLE 3:** Effects of supplemental zinc source and zinc level on the growth performance of broilers.

Item	Added Zn (mg/kg)	Day 1–7				Day 8–14				Day 1–14		
		BW on day 7 (g)	AFI (g/bird)	BWG (g/bird)	FCR (g:g)	BW on day 14 (g)	AFI (g/bird)	BWG (g/bird)	FCR (g:g)	AFI (g/bird)	BWG (g/bird)	FCR (g:g)
Control^a^	0	132.1	132.6^b^	85.5	1.55^b^	323.1	333.3	191.8	1.75	463.0	276.4	1.68
ZnSO_4_ ^c^	40	120.7	97.2	74.5	1.31^c^	304.3	292.0	183.5	1.59	389.2	258.2	1.51^c^
	80	125.8	112.9	79.2	1.46^ab^	310.2	280.2	182.5	1.59	421.1	278.5	1.51^c^
	120	122.2	111.0	75.8	1.46^ab^	298.2	294.0	180.5	1.67	405.0	251.8	1.61^abc^
Zn-Met^a^	40	126.9	110.7	80.5	1.40^bc^	320.5	337.2	193.7	1.76	448.0	274.1	1.63^abc^
	80	128.6	124.1	82.1	1.51^a^	333.2	373.5	204.6	1.83	497.6	286.6	1.73^a^
	120	127.7	108.8	81.1	1.36^bc^	325.0	314.3	197.5	1.60	424.1	278.2	1.53^bc^
Zn-Gly^a^	40	129.1	108.7	82.5	1.34^c^	353.7	349.6	222.4	1.72	464.3	307.0	1.51^bc^
	80	136.0	114.7	89.9	1.34^c^	352.9	346.3	218.4	1.68	474.1	306.9	1.58^bc^
	120	127.0	112.8	87.2	1.31^c^	335.4	370.6	208.1	1.80	483.5	289.4	1.67^ab^
SEM		3.75	5.69	3.53	0.04	10.29	19.55	8.84	0.09	20.60	9.25	0.06
Zn Source^c^	ZnSO_4_	122.9	107.1	76.5^b^	1.43	304.3^c^	288.7^b^	182.2^c^	1.62	405.1^b^	262.9^c^	1.55
	Zn-Met	127.8	114.5	81.2^a^	1.43	326.3^b^	341.6^a^	198.6^b^	1.73	456.5^a^	279.7^b^	1.63
	Zn-Gly	130.7	112.1	86.5^a^	1.33	347.4^a^	355.5^a^	216.3^a^	1.73	474.0^a^	301.1^a^	1.59
SEM		2.21	3.28	2.09	0.02	5.81	10.81	4.99	0.05	11.79	5.23	0.04
Added Zn^c^	40	125.6	105.5	79.2	1.35	326.3	326.3	194.9	1.69	433.8	279.8	1.55
(mg/kg)	80	130.1	117.2	83.8	1.44	332.1	333.3	201.4	1.70	464.3	290.7	1.61
	120	125.6	110.9	81.4	1.39	319.6	326.3	195.4	1.69	437.5	273.2	1.60
SEM		2.16	3.28	2.11	0.02	5.69	10.30	5.88	0.04	10.86	5.11	0.03
*p*-value												
Zn-Source		0.050	0.264	0.008	0.002	<0.001	<0.001	<0.001	0.171	<0.001	<0.001	0.162
Added Zn		0.253	0.051	0.310	0.013	0.310	0.859	0.639	0.985	0.131	0.067	0.429
Interaction		0.876	0.562	0.943	0.003	0.857	0.158	0.823	0.263	0.299	0.663	0.042

BW, body weight; AFI, average feed intake; BWG, body weight gain; FCR, the ratio of feed consumption and body weight gain; Zn-Met, zinc-methionine chelate; Zn-Gly, zinc-glycine chelate; and SEM, standard error of mean.

^a^ Data are mean values of eight replicate pens per treatment.

^b^ Different from all Zn supplemental groups (*p* < 0.05).

^c^ Data are mean values of 24 replicate pens per treatment.

^a-c^ Means without the same superscript letters in the same column differ (*p* < 0.05).

Dietary Zn addition, as a whole, had no significant effects on the BW of broilers on day 14, and the AFI, BWG, and FCR (*p* > 0.15) compared with the control during days 8–14. In all Zn-added treatments, the BW on day 14 and BWG during days 8–14 were higher in the Zn-Gly treatment than those in the Zn-Met treatment (*p* < 0.05), which were higher than those in the ZnSO_4_ treatment (*p* < 0.05). The Zn-Met and Zn-Gly treatments also had higher AFI during days 8–14 compared with the ZnSO_4_ treatment (*p* < 0.05). However, added Zn level and the interaction between added Zn source and Zn level had no significant effect on the AFI, BWG, and FCR during days 8–14 (*p* > 0.15).

In the overall period (days 1–14), dietary Zn addition, as a whole, had no significant effect on the AFI, BWG, and FCR (*p* > 0.10) compared with the control. In all Zn-added treatments, addition of Zn-Met or Zn-Gly increased (*p* < 0.05) the AFI and BWG compared with the ZnSO_4_ treatment, and the BWG was higher in the Zn-Gly treatment than in the Zn-Met treatment (*p* < 0.05) during days 1–14. No significant difference in FCR was observed among three added-Zn sources for 40 or120 mg Zn/kg treatments (*p* > 0.10), whereas this ratio was higher in the Zn-Met group than in the Zn-Gly or ZnSO_4_ group when 80 mg Zn/kg was given (*p* < 0.05).

### Zn concentrations in different tissues

The effects of supplemental Zn source and Zn level on the Zn content in different tissues of broilers are shown in Table 4. Overall, Zn supplementation significantly increased (*p* < 0.05) Zn concentrations in the liver, tibia, and pancreas of broilers on days 7 and 14 compared with the control. In all Zn-added groups, Zn-Met addition increased the tibia Zn level compared with the Zn-Gly or ZnSO_4_ treatment on day 7 (*p* < 0.05), whereas Zn sources did not affect it on day 14. Supplemental Zn sources also did not affect the Zn concentrations in the liver and pancreas on days 7 and 14 (*p* > 0.10). However, supplemental Zn level significantly affected Zn concentration in different tissues of broilers on days 7 and 14, except for pancreas Zn on day 7. Addition of 120 mg Zn/kg increased the liver Zn concentration compared with 40 or 80 mg/kg Zn addition treatments (*p* < 0.05). Likewise, the tibia Zn concentration increased significantly (*p* < 0.05) with increasing supplemental Zn level from 40 to 120 mg/kg Zn on day 7. On day 14, the concentrations of Zn in the liver, tibia, and pancreas increased linearly with increasing supplemental Zn level (*p* < 0.001). However, dietary supplemental Zn source and Zn level did not have interactive effect on the Zn content in the liver, tibia, and pancreas of broilers on days 7 and 14.

**TABLE 4:** Effects of supplemental zinc source and zinc level on zinc concentration in different tissues of broilers.

Item	Added Zn (mg/kg)	Day 7			Day 14		
		Liver^a^ (μg/g)	Tibia^b^ (μg/g)	Pancreas^a^ (μg/g)	Liver^a^ (μg/g)	Tibia^b^ (μg/g)	Pancreas^a^ (μg/g)
Control^c^	0	20.55^d^	176.3^d^	32.76^d^	8.91^d^	137.3^d^	24.14^d^
ZnSO_4_ ^c^	40	26.51	333.1	61.44	23.88	361.0	45.31
	80	29.24	364.8	63.43	26.61	418.7	46.22
	120	34.43	394.3	76.10	24.67	426.9	57.75
Zn-Met^c^	40	26.62	375.0	61.61	24.09	372.8	48.29
	80	24.58	397.9	75.87	25.25	421.5	51.53
	120	31.48	403.3	71.65	28.02	432.7	51.21
Zn-Gly^c^	40	24.96	343.6	67.86	22.69	361.4	42.27
	80	25.42	394.4	67.86	26.78	415.2	54.13
	120	34.55	380.5	70.71	27.94	420.3	54.95
SEM		1.87	17.03	4.45	1.39	24.74	3.18
Zn-source^e^	ZnSO_4_	30.06	364.0^b^	66.99	25.05	402.2	49.76
	Zn-Met	27.56	392.1^a^	69.71	25.79	409.0	50.34
	Zn-Gly	28.31	357.9^b^	68.81	25.81	399.0	50.45
SEM		1.06	10.15	2.88	0.84	14.38	1.85
Added Zn^e^	40	26.03^b^	350.5^c^	63.64	23.55^b^	365.0^b^	45.29^b^
(mg/kg)	80	26.41^b^	370.7^b^	69.05	26.21^a^	418.5^a^	50.63^ab^
	120	33.49^a^	392.7^a^	72.82	26.88^a^	426.6^a^	54.63^a^
SEM		1.07	9.59	2.89	0.84	13.68	1.81
*p*-value							
Zn-source		0.263	0.043	0.793	0.801	0.883	0.962
Added Zn		<0.001	0.012	0.099	0.017	0.006	0.002
Interaction		0.578	0.812	0.334	0.509	0.991	0.162
Linear^f^		<0.001	<0.001	<0.001	<0.001	<0.001	<0.001

ZnSO_4_, zinc sulfate heptahydrate; Zn-Met, zinc methionine chelate; Zn-Gly, zinc-glycine chelate; and SEM, standard error of mean.

^a^ Fresh tissue basis.

^b^ Ash weight basis.

^c^ Data are mean values of eight replicate pens per treatment.

^d^ Different from all Zn supplemental groups (*p* < 0.05).

^e^ Data are mean values of 24 replicate pens per treatment.

^f^ Linear effect of the added Zn level.

^a-c^ Means without the same superscript letters in the same column differ (*p* < 0.05).

### Pancreas *MT* expression

The effects of supplemental Zn source and Zn level on MT concentration and *MT* mRNA abundance in the pancreas of broilers are shown in Table 5. Overall, Zn supplementation significantly increased (*p* < 0.05) the MT protein concentration and *MT* mRNA abundance in the pancreas of broilers on days 7 and 14. In all Zn-added groups, the pancreas MT protein level was higher in the Zn-Met treatment than that in the ZnSO_4_ treatment (*p* < 0.05) on day 7, and it was intermediate for the Zn-Gly treatment. Added Zn source did not significantly affect the *MT* mRNA abundance in the pancreas of broilers on day 7 (*p* > 0.05). The pancreas MT concentration and *MT* mRNA abundance increased linearly with increasing supplemental Zn level (*p* < 0.05) by tendency analysis, whereas the difference in pancreas *MT* mRNA abundance between 40 and 80 mg/kg Zn addition treatments did not attain the significant level (*p* > 0.05).

**TABLE 5:** Effects of supplemental Zn source and zinc level on the concentration and mRNA abundance of metallothionine in the pancreas of broilers.

Item	Added Zn mg/kg	Day 7		Day 14	
		MT (ng/g protein)	*MT* mRNA (fold-change)	MT (ng/g protein)	*MT* mRNA (fold-change)
Control^a^	0	3.35^b^	1.01^b^	1.99^b^	1.04^b^
ZnSO_4_ ^a^	40	3.93	1.30	2.07	2.09
	80	4.61	1.41	2.44	3.06
	120	7.58	1.95	2.43	3.69
Zn-Met^a^	40	4.59	1.32	2.19	2.99
	80	8.74	1.53	2.46	4.01
	120	9.51	2.20	3.33	6.38
Zn-Gly^a^	40	4.29	1.35	2.14	1.95
	80	6.19	1.42	2.35	2.75
	120	9.24	1.95	2.85	3.59
SEM		0.78	0.20	0.21	0.37
Zn-source^c^	ZnSO_4_	5.37^b^	1.55	2.31	2.95^b^
	Zn-Met	7.61^a^	1.68	2.66	4.46^a^
	Zn-Gly	6.58^ab^	1.57	2.45	2.76^b^
SEM		0.45	0.11	0.13	0.20
Added Zn^c^	40	4.27^c^	1.32^b^	2.13^b^	2.34^c^
(mg/kg)	80	6.51^b^	1.45^b^	2.41^b^	3.27^b^
	120	8.78^a^	2.03^a^	2.87^a^	4.55^a^
SEM		0.49	0.10	0.12	0.24
*p*-value					
Zn-source		0.009	0.668	0.217	<0.001
Added Zn		<0.001	<0.001	0.001	<0.001
Interaction		0.289	0.968	0.472	0.077
Linear^d^		<0.001	<0.001	<0.001	<0.001

MT, metallothionein; ZnSO_4_, zinc sulfate heptahydrate; Zn-Met, zinc-methionine chelate; and Zn-Gly, zinc-glycine chelate.

^a^ Data are mean values of eight replicate pens per treatment.

^b^ Different from all Zn supplemental groups (*p* < 0.05).

^c^ Data are mean values of 24 replicate pens per treatment.

^d^ Linear effect of the added Zn level.

^a-c^ Means without the same superscript letters in the same column differ (*p* < 0.05).

On day 14, the Zn-Met treatment increased pancreas *MT* mRNA abundance compared with the Zn-Gly or ZnSO_4_ treatment (*p* < 0.001), whereas added Zn sources did not significantly affect the pancreas MT concentration (*p* > 0.10). The pancreas MT concentration and *MT* mRNA abundance increased linearly with increasing supplemental Zn level (*p* < 0.05), while no significant difference in pancreas MT concentration between 40 and 80 mg Zn/kg addition treatments on day 14 was observed.

### Relative bioavailability of Zn from Zn-Gly or Zn-Met chelate

Different biological indices were regressed on the supplemental Zn intake and significant multiple linear regression relationships were observed (*p* < 0.001) for the tibia and pancreas Zn concentrations, pancreas MT content, and pancreas *MT* mRNA abundance in broilers at 7 and 14 days of age (Table 6). Thus, the RBV was calculated using ZnSO_4_ as a standard (100%) by means of slope ratio methodology and the results are shown in Table 7. On day 7, no significant differences in the RBV were observed among three supplemental Zn sources, assessed by tibia Zn, pancreas Zn, and pancreas *MT* mRNA (*p* > 0.40), whereas it was in the following order: Zn-Met > Zn-Gly > ZnSO_4_ (*p* < 0.05), assessed by the pancreas MT content. The bioavailable Zn from Zn-Met or Zn-Gly was 1.73 or 1.44 times that from ZnSO_4_, respectively, and the bioavailable Zn from Zn-Met was 1.20 times that from Zn-Gly. On day 14, no significant differences were also observed in the RBV of Zn among three supplemental Zn sources based on tibia Zn and pancreas Zn (*p* > 0.46). However, the RBV of Zn was in the order Zn-Met > Zn-Gly > ZnSO_4_ (*p* < 0.05), assessed by the pancreas MT content. The bioavailable Zn from Zn-Met or Zn-Gly was 1.75 or 1.39 times that from ZnSO_4_, respectively, and the bioavailable Zn from Zn-Met was 1.25 times that from Zn-Gly. The RBV of Zn were significantly higher for Zn-Met than that for Zn-Gly or ZnSO_4_ (*p* < 0.05) based on the pancreas *MT* mRNA expression, and there was no significant difference between Zn-Gly and Zn-Met (*p* > 0.10).

**TABLE 6:** Multiple linear equation of different Zn sources^a^.

Age	Dependent variable	Regression equation^b^	*R* ^2^	*p*-value
Day 7	Tibia Zn	Y = 257 + 11.70X_1_ +13.55X_2_ + 10.01X_3_	0.739	<0.001
	Pancreas Zn	Y = 57.7 + 1.088X_1_ +1.365X_2_ + 1.077X_3_	0.598	<0.001
	Pancreas MT	Y = 2.57 + 0.312X_1_ +0.556X_2_ + 0.451X_3_	0.620	<0.001
	Pancreas *MT* mRNA	Y = 0.97 + 0.066X_1_ +0.074X_2_ + 0.065X_3_	0.710	<0.001
Day 14	Tibia Zn	Y = 348 + 1.578X_1_ +1.730X_2_ + 1.348X_3_	0.678	<0.001
	Pancreas Zn	Y = 37.1 + 0.257X_1_ +0.294X_2_ + 0.259X_3_	0.421	<0.001
	Pancreas MT	Y = 1.79 + 0.059X_1_ +0.093X_2_ + 0.074X_3_	0.747	<0.001
	Pancreas *MT* mRNA	Y = 1.14 + 0.076X_1_ +0.121X_2_ + 0.068X_3_	0.579	<0.001

MT, metallothionein.

^a^ The regression analyses were based on all observations in different treatments (n = 8 for each treatment).

^b^ Y represents respective dependent variable, including tibia Zn (μg/g, ash basis), pancreas Zn (μg/g, fresh basis), pancreas MT (ng/g protein), or pancreas MT mRNA abundance (relative fold-change); X_1_, X_2_, and X_3_ represent supplemental Zn intake (mg) from zinc sulfate (ZnSO_4_), zinc-bismethionate (Zn-Met), and zinc-bisglycinate (Zn-Gly), respectively.

**TABLE 7:** Relative bioavailability values (RBVs) of zinc based on the multiple regression of different biological indices on supplemental zinc intake.

Age	Dependent variable	Zn source	Regression coefficient^a^		RBV^a^		*p*-value^b^
			Slope	Se	%	Se	
Day 7	Tibia Zn	ZnSO_4_	11.70	2.05	100		0.432
		Zn-Met	13.55	2.04	116	14.6	
		Zn-Gly	10.00	1.89	85	20.8	
	Pancreas Zn	ZnSO_4_	1.088	0.465	100		0.653
		Zn-Met	1.365	0.473	125	13.2	
		Zn-Gly	1.077	0.441	99	14.3	
	Pancreas MT	ZnSO_4_	0.312^c^	0.069	100		0.021
		Zn-Met	0.556^a^	0.071	173	15.1	
		Zn-Gly	0.451^b^	0.072	144	12.8	
	Pancreas *MT* mRNA	ZnSO_4_	0.065	0.011	100		0.746
		Zn-Met	0.074	0.010	113	12.3	
		Zn-Gly	0.065	0.014	99	16.2	
Day14	Tibia Zn	ZnSO_4_	1.578	0.613	100		0.468
		Zn-Met	1.730	0.562	110	10.6	
		Zn-Gly	1.348	0.674	85	14.1	
	Pancreas Zn	ZnSO_4_	0.257	0.045	100		0.683
		Zn-Met	0.294	0.052	115	14.8	
		Zn-Gly	0.259	0.043	101	13.6	
	Pancreas MT	ZnSO_4_	0.059^c^	0.019	100		0.032
		Zn-Met	0.093^a^	0.025	175	15.7	
		Zn-Gly	0.074^b^	0.022	139	14.8	
	Pancreas *MT* mRNA	ZnSO_4_	0.076^b^	0.010	100		0.040
		Zn-Met	0.121^a^	0.014	158	15.9	
		Zn-Gly	0.068^b^	0.010	90	10.2	

MT, metallothionein; ZnSO_4_, zinc sulfate; Zn-Met, zinc-bismethioninate chelate; and Zn-Gly, zinc-bisglycinate chelate.

^a^ The regression coefficients were based on all observations in each treatment (n = 8).

^b^ The relative bioavailability value (RBV) of zinc from Zn-Met or Zn-Gly was calculated by the ratio of slope (multiple linear regression) using ZnSO_4_ as a standard (100%).

^c^ *p*-value for the difference in slope among Zn sources.

a-c Means without the same superscript letters in the same column differ (*p* < 0.05) for the respective dependent variable.

## Discussion

The term “bioavailability” is defined as the degree to which an ingested nutrient in a particular source is absorbed in a form that can be utilized in metabolism by animal (Ammerman et al., 1995). The growth performance can be used as a response biomarker to assess the RBV of Zn for broilers when dietary graded Zn levels are below their requirement in the purified diet (Wedekind and Baker, 1990; Wedekind et al., 1992). However, in this study, all Zn-supplemented treatments did not increase the BWG of starter broilers during days 1–14 compared with the control, and no significant differences occurred in AFI and BWG among the Zn-supplemented treatments. These results indicated that the addition of 40 mg/kg Zn in the corn-soybean meal diet is sufficient for the growth of starter broilers (Huang et al., 2007; Bao et al., 2009). No negative effect of the control diet on the BWG compared with the Zn-supplemented treatments presented herein might be due to the increase of feed intake during days 1–7. Our result of no increase in the growth performance with increasing dietary Zn level proved that it is not a suitable biomarker to assess the RBV of Zn for the broilers when dietary Zn level is higher than their requirement (Wedekind et al., 1992) because the growth performance of chicks can be affected by many dietary factors other than Zn (Moran, 2017). In addition, our study found that Zn-Gly and Zn-Met increased the BWG and AFI compared with ZnSO_4_ during days 8–14. This finding might be due to the important role of Zn in the appetite regulation for broilers (Dewar et al., 1982). The increase of the BWG in the Zn-Gly treatment compared with the Zn-Met treatment might be attributed to the dissociated glycine from Zn-Gly chelate at intestinal mucosal barrier (Ammerman et al., 1995), which could improve mucosal absorption function in the small intestine of poultry (Ospina-Rojas et al., 2013; Moran, 2017).

The Zn concentrations in different tissues, such as the tibia, liver, pancreas, kidney, and intestinal mucosa, have been used as the biological criterion for RBV assays with the poultry (Sandoval et al., 1997; Cao et al., 2000; Huang et al., 2009). In the present study, we only chose the Zn concentration in the tibia, liver, and pancreas of broilers to determine for the RBV assay, because the mucosal Zn accumulation only reflects the status of Zn absorption and homeostasis (Cao et al., 2000) and uptake of Zn by kidney is affected by the feed intake for broilers (Sandoval et al., 1997; Sandovel et al., 1999). Our results demonstrated that the tibia and pancreas accumulated more Zn than the liver, and the pancreas Zn level was higher than that of tibia Zn if on the wet-tissue basis, which were consistent with the previous findings (Sandoval et al., 1997; Sandovel et al., 1998). Moreover, we did not find the differences in pancreas Zn and liver Zn on day 7 and Zn concentrations in the tibia, pancreas, and liver on day 14 among Zn-Met, Zn-Gly, and ZnSO_4_ treatments. Our study also found that the RBV of Zn as Zn-Met was comparable with that as Zn-Gly or ZnSO_4_ evaluated by tibia and pancreas Zn levels. In agreement, Suo et al. (2015) reported that the RBV of Zn from Zn-Met was just as bioavailable as that from ZnSO_4_ based on multiple linear regression ratio of pancreas or tibia Zn on dietary Zn intake. Similarly, the estimated RBV of Zn from a certain Zn-amino acid chelate did not differ that from ZnSO_4_ based on tibia Zn (Cao et al., 2000; Huang et al., 2009). However, the RBV of Zn from Zn-Met was higher in the purified (crystalline amino acid) or semipurified (soy isolate) diets compared to that from ZnSO_4_ in broilers given the Zn level was far below the requirement (Wedekind et al., 1992). The discrepancies in these findings might be due to the differences in the composition of the basal diet and supplemental Zn level.

MT plays a crucial role in the cellular Zn homeostasis because it exhibits high affinity binding to Zn ion in cytoplasm (Kimura and Kambe, 2016). Zn intake has been shown to induce tissue MT synthesis (Kimura and Itoh, 2008). The present results found that the concentration and mRNA abundance of pancreas MT increased linearly as dietary supplemental Zn level increased. In agreement, pancreas MT protein or *MT* mRNA expression increased linearly in chicks with increasing dietary Zn level up to subtherapeutic level (1,000 or 1,200 mg/kg) for chicks (Sandovel et al., 1998). These results confirmed that pancreas MT expression can be used as a biomarker to assess the RBV of Zn for broiler chickens feeding excess dietary Zn (Sandoval et al., 1997; Sandovel et al., 1998). In the present study, the results of multiple regression slope ratio of the pancreas MT concentration on dietary supplemental Zn intake showed the RBV of Zn was in the following order: Zn-Met > Zn-Gly > ZnSO_4_. However, Zn-Met and other unknown Zn-amino acid complexes had bioavailability values similar to that of ZnSO_4_ for chicks with dietary supplementation of 200–600 mg/kg Zn (Cao et al., 2000). Cao et al. (2002) reported that the RBV of Zn from Zn-Met was 0.78–0.91 of Zn-acetate standard (1.00) for starter broilers given 30–90 mg Zn/kg diet, based on the intestinal mucosal MT. Huang et al. (2009) also found that the unknown Zn-amino acid chelate had a comparable RBV of Zn relative to ZnSO_4_ assessed by pancreas MT. The differences in the standard Zn source, the selected specific tissue for the MT determination, and the ligands of Zn-amino acid chelate might explain the discrepancies in these studies. In addition, the responses of pancreas MT to dietary supplemental Zn intake presented herein were inconsistent with the aforementioned findings of the RBV based on tissue Zn accumulation, which might be due to the fact that the response of MT is more sensitive to the Zn uptake than tissue Zn accumulation (Baltaci et al., 2018; Zhao et al., 2019). These results indicated the pancreas MT concentration was more suitable to evaluate the RBV of Zn from different sources for starter broilers than the tissue Zn accumulation.

Several studies found that tissue *MT* mRNA could be used as a response criterion to evaluate the RBV of Zn from different Zn sources in chickens (Cao et al., 2002; Huang et al., 2009). In the present study, we found that the RBV of Zn from Zn-Met did not differ from that as Zn-Gly or ZnSO_4_ based on pancreas *MT* mRNA abundance on day 7, and it was lower assessed by pancreas *MT* mRNA abundance than pancreas MT concentration. The results were not in line with the previous finding that the *MT* mRNA abundance in pancreas was more sensitive in reflecting differences in bioavailability among organic Zn sources than the MT concentration in pancreas (Huang et al., 2009). There were two potential reasons to explain these discrepancies. First, the accumulation of *MT* mRNA may be partly regulated by *MT* mRNA stability, which relates to the concentrations of metals (Zn and Cd) and inflammation factors (De et al., 1991; Cousins and Lee-Ambrose, 1992). Second, the increase of MT protein can cause the decrease of the free Zn ion, resulting in the downregulation of *MT* transcription (Kimura and Itoh, 2008). Additionally, we found that the RBV of Zn from Zn-Met or Zn-Gly compared with ZnSO_4_ used as a standard on day 7 was similar to that on day 14, based on the pancreas MT concentration. However, based on the pancreas *MT* mRNA abundance, the RBV of Zn from Zn-Met or Zn-Gly on day 7 differed from that on day 14. These results indicated that the pancreas MT concentration was more stable to evaluate the RBV of Zn as compared with the pancreas *MT* mRNA abundance. Future work should focus on the potential mechanisms underlying the inconsistent expression of *MT* mRNA and protein in the pancreas of broilers for the Zn bioavailability assay.

## Conclusion

The Zn concentrations in the tibia, pancreas, and liver, pancreas MT content, and pancreas *MT* mRNA abundance of starter broilers (d 1–14) increased linearly with increasing supplemental Zn from 40 to 120 mg/kg for the corn-soybean meal basal diet. Zn-Met had similar bioavailable Zn to Zn-Gly, based on pancreas or tibia Zn, whereas it had higher levels of bioavailable Zn than Zn-Gly, using the pancreas MT concentration as a response biomarker, for the starter broilers.

## Data Availability

The original contributions presented in the study are included in the article/supplementary material; further inquiries can be directed to the corresponding author.
